# Supplementary figures and images for: A preoperative predictive study of advantages of airway changes after maxillomandibular advancement surgery using computational fluid dynamics analysis
Source: PLoS One. 2021 Aug 11;16(8):e0255973. doi: 10.1371/journal.pone.0255973 (PMC8357109; doi:10.1371/journal.pone.0255973)

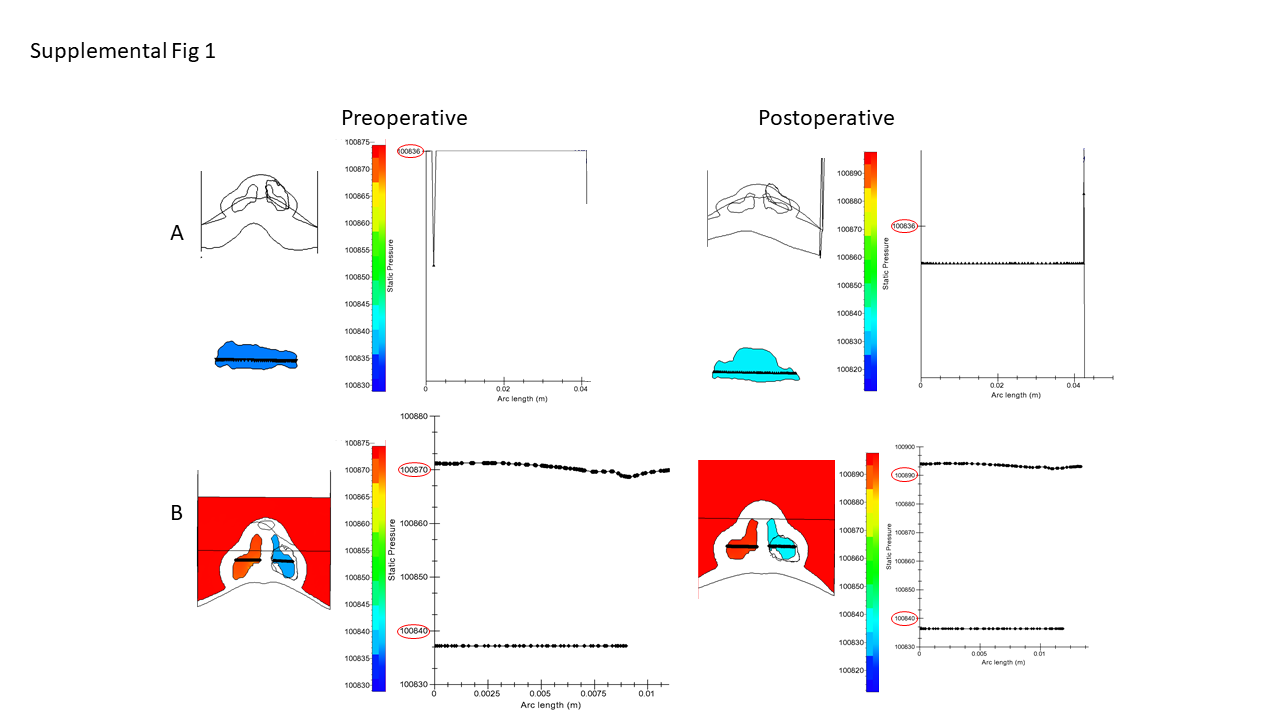

Supplement: S1 Fig — Preoperative and postoperative static pressure on the outlet (A) and inlet (B) in the right nasal passage for the Case I patient. (TIF) [file pone.0255973.s001.tif]

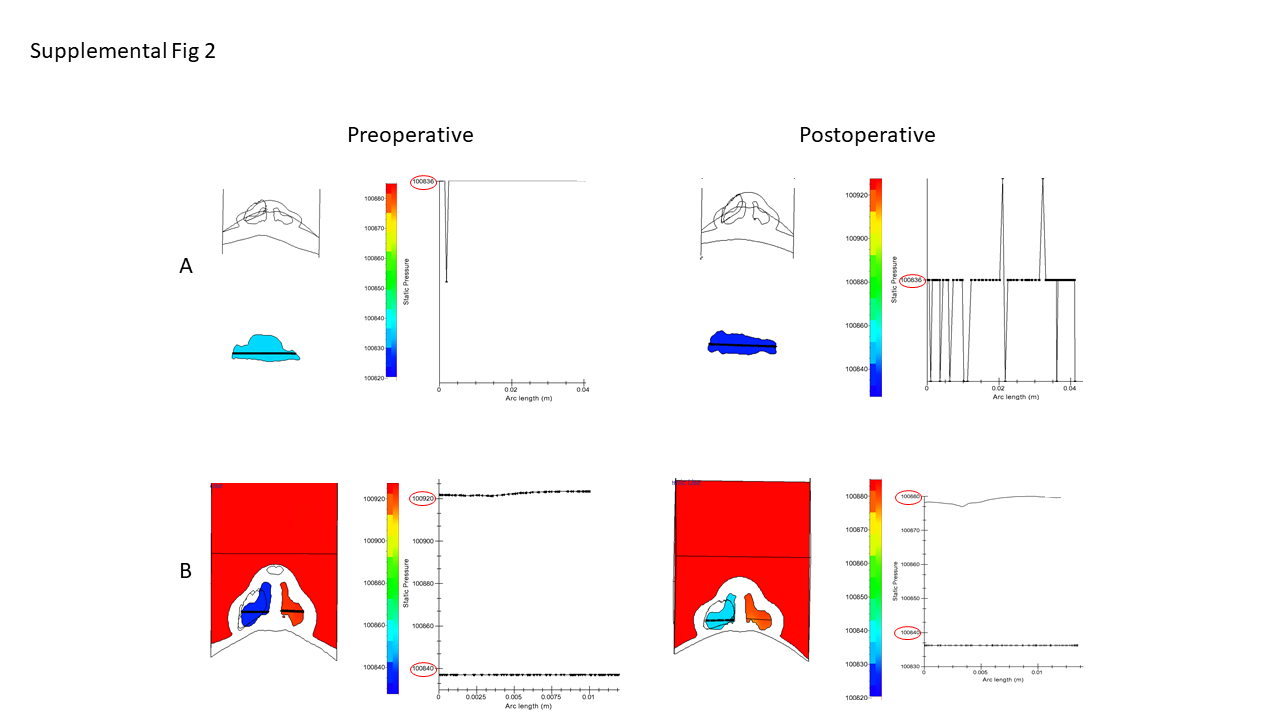

Supplement: S2 Fig — Preoperative and postoperative static pressure on the outlet (A) and inlet (B) in the left nasal passage for the Case I patient. (TIF) [file pone.0255973.s002.tif]
