# Supplementary material for: A preoperative predictive study of advantages of airway changes after maxillomandibular advancement surgery using computational fluid dynamics analysis
Source: PLoS One. 2021 Aug 11;16(8):e0255973. doi: 10.1371/journal.pone.0255973 (PMC8357109; doi:10.1371/journal.pone.0255973)
Supplement: Attachment — Submitted filename: rebuttal letter_PLOS ONE.docx [file pone.0255973.s003.docx]

July 23, 2021

Dr. Emily Chenette and Giannicola Iannella

Editors-in-Chief and Academic Editor

*PLOS One*

Dear Editor:

We are grateful for the review of our paper (Ms. Ref. No.: PONE-D-21-18930) entitled “**A preoperative predictive study of advantages of airway changes after maxillomandibular advancement surgery using computational fluid dynamics analysis**”, and now submit the revised version of the manuscript in which we have addressed the journal requirements and the concerns of the reviewer.

**Journal Requirements**

1. Please ensure that your manuscript meets PLOS ONE's style requirements, including those for file naming.

***Response:***

We have confirmed it.

2. Please review your reference list to ensure that it is complete and correct.

***Response:***

We have double checked that the reference had been complete and correct.

3. You indicated that you had ethical approval for your study. In your Methods section, please ensure you have also stated whether you obtained consent from parents or guardians of the minors included in the study or whether the research ethics committee or IRB specifically waived the need for their consent.

***Response:***

We have added the sentence ‘Informed consent was obtained from parents/guardians of subjects < 20 years of ages.’ in the Materials and methods section (page 4, lines 86).

4. Thank you for stating the following in the Acknowledgments Section of your "We wish to thank Editage (www.editage.jp) for English language editing. This study was supported by the Japan Society for the Promotion of Science (KAKENHI, no. 19K10294) grants from the Dental Research Center (2019, 2020, and 2021) and Sato Fund (2018), Nihon University School of Dentistry (Tokyo, Japan)."

We note that you have provided funding information that is not currently declared in your Funding Statement. However, funding information should not appear in the Acknowledgments section or other areas of your manuscript. We will only publish funding information present in the Funding Statement section of the online submission form.

Please remove any funding-related text from the manuscript and let us know how you would like to update your Funding Statement. Currently, your Funding Statement reads as follows:

"This study was supported by the Japan Society for the Promotion of Science (KAKENHI, no. 19K10294) grants from the Dental Research Center (2019, 2020 and 2021) and Sato Fund (2018), Nihon University School of Dentistry (Tokyo, Japan)."

Please include your amended statements within your cover letter; we will change the online submission form on your behalf.

***Response:***

We have deleted the sentence "This study was supported by the Japan Society for the Promotion of Science (KAKENHI, no. 19K10294) grants from the Dental Research Center (2019, 2020, and 2021) and Sato Fund (2018), Nihon University School of Dentistry (Tokyo, Japan)." in the Acknowledgments Section and instead we would like you to move it into the Funding Statement section.

5. PLOS requires an ORCID iD for the corresponding author in Editorial Manager on papers submitted after December 6th, 2016. Please ensure that you have an ORCID iD and that it is validated in Editorial Manager. To do this, go to ‘Update my Information’ (in the upper left-hand corner of the main menu), and click on the Fetch/Validate link next to the ORCID field. This will take you to the ORCID site and allow you to create a new iD or authenticate a pre-existing iD in Editorial Manager. Please see the following video for instructions on linking an ORCID iD to your Editorial Manager account: <https://www.youtube.com/watch?v=_xcclfuvtxQ>

***Response:***

I have linked my ORCID id to Editorial Manager account.

6. We note that Figure(s) 1 and 2 in your submission contain copyrighted images. All PLOS content is published under the Creative Commons Attribution License (CC BY 4.0), which means that the manuscript, images, and Supporting Information files will be freely available online, and any third party is permitted to access, download, copy, distribute, and use these materials in any way, even commercially, with proper attribution. For more information, see our copyright guidelines: http://journals.plos.org/plosone/s/licenses-and-copyright.

We require you to either (1) present written permission from the copyright holder to publish these figures specifically under the CC BY 4.0 license, or (2) remove the figures from your submission:

a. You may seek permission from the original copyright holder of Figure(s) 1 and 2 to publish the content specifically under the CC BY 4.0 license.

We recommend that you contact the original copyright holder with the Content Permission Form (http://journals.plos.org/plosone/s/file?id=7c09/content-permission-form.pdf) and the following text:

“I request permission for the open-access journal PLOS ONE to publish XXX under the Creative Commons Attribution License (CCAL) CC BY 4.0 (http://creativecommons.org/licenses/by/4.0/). Please be aware that this license allows unrestricted use and distribution, even commercially, by third parties. Please reply and provide explicit written permission to publish XXX under a CC BY license and complete the attached form.”

Please upload the completed Content Permission Form or other proof of granted permissions as an "Other" file with your submission.

In the figure caption of the copyrighted figure, please include the following text: “Reprinted from [ref] under a CC BY license, with permission from [name of publisher], original copyright [original copyright year].”

b. If you are unable to obtain permission from the original copyright holder to publish these figures under the CC BY 4.0 license or if the copyright holder’s requirements are incompatible with the CC BY 4.0 license, please either i) remove the figure or ii) supply a replacement figure that complies with the CC BY 4.0 license. Please check copyright information on all replacement figures and update the figure caption with source information. If applicable, please specify in the figure caption text when a figure is similar but not identical to the original image and is therefore for illustrative purposes only.

***Response:***

We truly apologize confusing you because of our inappropriate expression. Figures 1 and 2 are our original data of our eight patients. That means these are not copyrighted images. As we re-wrote correctly, please check it again.

**Reviewer’s comments**

**Reviewer: 1**

***Comments to the Author:***

The manuscript is well written, however minor corrections are needed to improve the quality.

***Comments:***

1. Introduction

line 44, It has been shown that obstructive sleep apneas are also correlated with different comoribidities compared to the classic cardiovascular ones. In fact, a correlation has recently been reported between OSAS and nasal disorders both sensory with altered smell and inflammation the alteration of different demonstrable biomarkers such as oxygen free radicals, cell free DNA or lipid peroxidation. please cite the paper DOI:10.1007/s00405-020-06316-w and DOI:10.3390/jcm10020277

***Response:***

We would like to thank the reviewer 1 who has reviewed our manuscript favorably and we appreciate the comments and suggestions to improve the content. As it has been suggested by the reviewer, we have made correction in the part of Introduction of manuscript.

2. Methods

the methods used correct and well structured.

The results are consistent with the methods set out.

Figures are really interesting, representing well what the results presuppose.

Discussion correct and inherent.

***Response:***

We appreciate the comments.

**Reviewer: 2**

***Comments to the Author:***

Interesting paper, only few suggestions to improve the structure and make it more fascinable:

***Comments:***

1. Introduction, after line 49:

In the case of a retrolingual obstruction it is not always necessary to intervene through mandibular advancement, especially if there is a lymphatic lingual base hypertrophy or a primary epiglottis that can be modeled through a supraglottoplasty. please cite DOI:10.1002/rcs.2106

A comparative stress test demonstrated excellent distribution of palatal vector forces after barbed surgery, reallocating and shaping the soft tissues in order to avoid collapsing of the walls. please cite DOI:10.1007/s00405-020-05883-2

***Response:***

We would like to thank the reviewer 2 who has reviewed our manuscript favorably and we appreciate the comments and suggestions to improve the content. As it has been suggested by the reviewer, we have made correction in the part of Introduction of manuscript..

2. Methods and Results really fascinating as the figures.

Good job!

***Response:***

We appreciate the comments.

Thank you for your review, and for the positive feedback on the quality of the writing of this report.

I look forward to hearing from you.

Sincerely,

Keiji Shinozuka

Department of Oral and Maxillofacial Surgery, Nihon University School of Dentistry

Tokyo, Japan

E-mail: [shinozuka.keiji@nihon-u.ac.jp](mailto:shinozuka.keiji@nihon-u.ac.jp)
